# Symptomatic and Asymptomatic Neurological Complications of Infective Endocarditis: Impact on Surgical Management and Prognosis

**DOI:** 10.1371/journal.pone.0158522

**Published:** 2016-07-11

**Authors:** Christine Selton-Suty, François Delahaye, Pierre Tattevin, Claire Federspiel, Vincent Le Moing, Catherine Chirouze, Pierre Nazeyrollas, Véronique Vernet-Garnier, Yvette Bernard, Sidney Chocron, Jean-François Obadia, François Alla, Bruno Hoen, Xavier Duval, AEPEI (Association pour l'Etude et la Prévention de l'Endocardite Infectieuse)

**Affiliations:** 1 Cardiologie, CHU Nancy-Brabois, 54511, Nancy, France; 2 Hôpital Cardiologique Louis Pradel, Hospices Civils de Lyon; Université Claude Bernard, Lyon, France; 3 Maladies Infectieuses et Réanimation Médicale, Centre Hospitalier Universitaire, Rennes, France; 4 Cardiologie, Centre Hospitalier Départemental Vendée, La Roche sur Yon, France; 5 Maladies infectieuses et tropicales, Centre Hospitalier Régional Universitaire; UMI 233 Institut de Recherche sur le Développement, Université de Montpellier, Montpellier, France; 6 UMR 6249 Laboratoire Chrono-environnement, université de Franche-Comté; Maladies infectieuses et tropicales, Centre Hospitalier Universitaire, Besançon, France; 7 Cardiologie, Centre Hospitalier Universitaire, Reims, France; 8 Microbiologie, Centre Hospitalier Universitaire, Reims, France; 9 Cardiologie, Centre Hospitalier Universitaire, Besançon, France; 10 Chirurgie Cardiaque, Centre Hospitalier Universitaire, Besançon, France; 11 Uninté INSERM 886 « cardioprotection », laboratoire de physiologie Lyon nord, UCBL1; Hôpital Louis Pradel–Chirurgie Cardiothoracique et Transplantation, Lyon, France; 12 Université de Lorraine, Université Paris Descartes, Apemac, EA4360; INSERM, CIC‐EC, CIE6, Nancy, France; 13 Maladies infectieuses et tropicales, Centre Hospitalier Universitaire, Point à Pitre, France; 14 Inserm CIC 1425, AP-HP, Hôpital Universitaire Bichat; Inserm U1137 IAME; Université Paris Diderot, Paris 7, UFR de Médecine-Bichat, Paris, France; Sapienza University of Rome, ITALY

## Abstract

**Objectives:**

Symptomatic neurological complications (NC) are a major cause of mortality in infective endocarditis (IE) but the impact of asymptomatic complications is unknown. We aimed to assess the impact of asymptomatic NC (AsNC) on the management and prognosis of IE.

**Methods:**

From the database of cases collected for a population-based study on IE, we selected 283 patients with definite left-sided IE who had undergone at least one neuroimaging procedure (cerebral CT scan and/or MRI) performed as part of initial evaluation.

**Results:**

Among those 283 patients, 100 had symptomatic neurological complications (SNC) prior to the investigation, 35 had an asymptomatic neurological complications (AsNC), and 148 had a normal cerebral imaging (NoNC). The rate of valve surgery was 43% in the 100 patients with SNC, 77% in the 35 with AsNC, and 54% in the 148 with NoNC (p<0.001). In-hospital mortality was 42% in patients with SNC, 8.6% in patients with AsNC, and 16.9% in patients with NoNC (p<0.001). Among the 135 patients with NC, 95 had an indication for valve surgery (71%), which was performed in 70 of them (mortality 20%) and not performed in 25 (mortality 68%). In a multivariate adjusted analysis of the 135 patients with NC, age, renal failure, septic shock, and IE caused by *S*. *aureus* were independently associated with in-hospital and 1-year mortality. In addition SNC was an independent predictor of 1-year mortality.

**Conclusions:**

The presence of NC was associated with a poorer prognosis when symptomatic. Patients with AsNC had the highest rate of valve surgery and the lowest mortality rate, which suggests a protective role of surgery guided by systematic neuroimaging results.

## Introduction

Symptomatic cerebral neurological complications (SNC) are a major cause of morbidity and mortality in patients with infective endocarditis (IE) and may interact with the decision for surgery.[1] Although not recommended by previous guidelines, systematic neuroimaging procedures (NIP) such as brain MRI or CT scan to look for asymptomatic cerebral neurological complications (AsNC) are often performed in patients with IE during everyday care. The discovery of such complications may impact the surgical management of the patient. For instance, as valve surgery is indicated in patients with large vegetations persisting after one or more symptomatic or silent embolic events, [2] the detection of an asymptomatic small ischemic event could lead to surgery. Conversely, the finding of a large hemorrhage may lead to postpone a surgical procedure.

However, the impact of the detection of AsNC on the prognosis of patients without clinical symptoms is not well known and differs according to studies. In a study by Thuny et al., a brain CT scan was performed systematically in 453 patients, 17 (4%) of whom had AsNC and a mortality rate that was not different from that of the 344 patients without neurological complication (NC) though lower than that of the 92 patients with SNC.[3] In a study by Duval et al., systematic brain MRI in IE patients showed a rate of NC as high as 80%, corresponding mostly to asymptomatic lesions. In addition, in this study, these findings impacted both diagnostic classification and patient's management plans, especially indication for valve surgery.[4] In the study by Cooper et al., on 40 patients with left-sided IE and brain MRI,[5] 70% had MRI evidence of subclinical brain embolization; 3 month-mortality was similar in the 13 patients with SNC and in the 19 patients with AsNC, but significantly higher than the mortality of the 8 patients with no cerebral lesions.

These different results regarding the impact of AsNC on mortality may partly be explained by the different sensitivity of CT scan and MRI in detecting asymptomatic NC and by the lack of statistical power.[6] Furthermore, the prognostic role of surgery among the various groups of patients according to their neurological status was not assessed.

Using our database originating from a large population-based study, our goals were: 1) to describe and compare the characteristics of patients with IE according to the presence or absence of SNC or AsNC as detected by systematic cerebral imaging performed in routine care and 2) to describe the relationship between NC, whether symptomatic or not, and valve surgery and outcome.

## Methods

### Patients and design

Among 938 notified patients, 497 cases of expert-validated definite IE were analyzed from a population-based observational survey conducted in 2008–2010 in France.[7] The design of this study and the type of data recorded have been described in details previously.[8] The study was approved by an institutional review committee (Comité de Protection des Personnes, Besançon, December 2007). Patients were informed of the study but did not have to provide individual consent, in accordance with French legal standards. Patient records/information was anonymized and de-identified prior to any analysis.

The present study is based on the 283 patients with definite left-sided IE who had at least one NIP (cerebral CT scan and/or MRI) performed as part of initial patient's evaluation.

#### Collection of neurological clinical and imaging data

Neurological complications were considered as symptomatic when patients presented with clinical signs (focal neurological deficit or decreased level of consciousness) or asymptomatic when patients had no clinical signs but abnormalities identified by systematic NIP. Neurological complications were classified as transient ischemic attack, permanent ischemic emboli, cerebral hemorrhage, brain abscess, or intracranial mycotic aneurysms according to the report of physicians in charge and to the results of NIP as interpreted by local radiologists. The diagnosis of meningitis was based on the clinical presentation and the presence of cerebrospinal fluid pleocytosis and/or positive bacterial cultures. Undefined cerebral events gathered patients with neurological symptoms that could not be classified in any of the other categories.

#### Surgical data

The indication for heart valve surgery during hospitalization was determined by the physicians in charge of the patient. For each patient, theoretical indication or lack of indication for surgery was prospectively recorded based on current guidelines using a predefined item list. Furthermore, the type of indication was classified as hemodynamic, infectious, embolic or a combination of these.[2] For each patient, the reasons for not performing surgery while theoretically indicated were recorded. Two different time intervals were calculated: 1) time from diagnosis to surgery was the time interval between the day of diagnosis and the day of surgery, 2) surgery timing was calculated as the time interval between surgical indication and surgery and was classified as ‘emergency’ if it was less than 48h, ‘elective’ if surgery was performed later, and ‘delayed’ if surgery was postponed because of a transitory contra-indication.

#### Mortality and follow-up

The in-hospital and one-year mortality rates were defined as the number of patients who died respectively during the initial hospital stay or during the year following the beginning of the initial hospital stay, whatever the cause of death, divided by the study population size. One-year all-cause mortality was collected from the patient's general practitioner, and when not available, from the French administrative registries and was obtained for all patients.

### Statistical analysis

Quantitative data are expressed as mean ± standard deviation and qualitative data as absolute and relative frequency. Comparison between different subgroups of patients was performed with the Chi-2 or the Fisher exact test for qualitative data and with the Student t test or the Wilcoxon test for quantitative data.

A first analysis of prognostic factors for in-hospital and 1-year mortality was performed among the whole population by a multivariate Cox model according to the neurological status with an adjustment for surgery. The variable "neurological complication" was categorized as absence of, symptomatic, or asymptomatic.

A second analysis of prognostic factors for in-hospital and 1-year mortality was performed among the patients with NC by bivariate and multivariate Cox models with an adjustment forced on valve surgery. Variables "septic shock" and "valvular surgery" were analyzed as time-dependent variables.

Results of Cox models are expressed as hazard ratios with a 95% confidence interval.

Survival estimates were computed by use of Kaplan-Meier methods. Log-rank tests were used to compare survival of patients according to the 3 categories of neurological complication. These were also performed among the operated or non-operated patients with or without surgical indication.

A p value less than 0.05 was considered significant. For multivariate models, a stepwise variable selection with a sle = 0.1 and sls = 0.05 was applied.

SAS 9.3 software was used for statistical analysis.

## Results

### Population

Among the 497 patients enrolled in the initial epidemiological study, 330 had at least one NIP (66%). Among the 399 with either left-sided or bilateral IE, 283 hat at least one NIP (71%). Characteristics of those 283 patients (106 male, mean age: 62.1±15.1 yrs) are displayed in Table 1. Of note, the 116 remaining patients who did not have systematic NIP had similar background characteristics than the patients without symptomatic NC (S1 Table).

**Table 1:** Patient characteristics according to the absence or the presence of neurological complications (NC) either asymptomatic or symptomatic.

	No NC (NoNC) N = 148	Any NC n = 135	p1	Asymptomatic NC (AsNC) n = 35	Symptomatic NC (SNC) n = 100	p2	p3 (NoNC vs AsNC)	p4 (NoNC vs SNC)
**Clinical and biological characteristics**								
Male gender	106 (71.6)	106 (78.5)	0.181	26 (74.3)	80 (80)	0.479	0.752	0.134
Age (years)	62.1 (±15.1)	61.1 (±15.8)	0.618	56.2 (±16.3)	62.9 (±15.4)	**0.032**	**0.045**	0.686
Chronic alcoholism	18 (12.8)	21 (15.9)	0.458	5 (14.7)	16 (16.3)	0.824	0.779	0.483
IV drug use	5 (3.4)	8 (5.9)	0.307	3 (8.6)	5 (5.0)	0.427	0.18	0.530
Smoking	32 (23)	25 (19.4)	0.467	8 (23.5)	17 (17.9)	0.476	0.95	0.344
Diabetes mellitus	34 (23)	26 (19.3)	0.445	7 (20.0)	19 (19.0)	0.897	0.704	0.454
Charlson score ≥1	96 (64.8)	81 (60.0)	0.398	17 (48.5)	64 (64.0)	0.109	0.075	0.889
Anticoagulation therapy	38 (25.7)	49 (36.3)	0.053	10 (28.6)	39 (39.0)	0.27	0.726	**0.026**
Aspirin therapy	24 (16.2)	28 (20.7)	0.326	5 (14.3)	23 (23.0)	0.274	0.779	0.181
*Underlying heart disease*			**0.015**			0.096	**0.046**	**0.016**
Native IE	124 ()	97 ()		26 ()	71 (71.0)			
Prosthetic IE	24 (16.2)	38 (28.1)		9 (25.7)	29 (29.0)			
*Type of valvular prosthesis*			**0.017**			0.303	0.408	**0.075**
Mechanical prosthesis	11 (7.4)	25 (18.5)		4 (11.4)	21 (21.0)			
Bioprosthesis, homograft, autograft	13 (8.8)	13 (9.6)		5 (14.3)	8 (8.0)			
*Presumed mode of acquisition*			0.276			0.096	**0.046**	0.762
Community	117 (80.1)	109 (85.2)		33 (94.3)	76 (81.7)			
Health-care associated	29 (19.9)	19 (14.8)		2 (5.7)	17 (18.3)			
*Complications*								
Embolic events (other than cerebral)	40 (27)	67 (49.6)	**<0.0001**	22 (62.9)	45 (45)	0.069	**<0.0001**	**0.003**
Septic shock (before surgery)	6 (4.1)	17 (12.6)	**0.009**	4 (11.4)	13 (13)	0.809	0.1	**0.009**
CRP > 120 mg/l	58 (40)	80 (62.8)	**0.0002**	15 (45.5)	66 (68.8)	**0.017**	0.565	**<0.0001**
**Cardiac lesions**								
Vegetation	136 (91.9)	126 (93.3)	**0.644**	32 (91.4)	94 (94.0)	0.695	1.00	0.530
*Vegetation size*			***0*.*009***			*0*.*623*	*0*.*184*	***0*.*008***
≤ 15 mm	82 (74.5)	58 (57.4)		16 (61.5)	42 (56)			
>15 mm	28 (25.5)	43 (42.6)		10 (38.5)	33 (44)			
Intracardiac abscess (at echo)	26 (17.6)	32 (23.7)	0.202	13 (37.1)	19 (19)	**0.03**	**0.011**	0.774
Significant valvular regurgitation	74 (50)	50 (37.9)	**0.042**	20 (57.1)	30 (30.9)	**0.006**	0.447	**0.003**
LVEF † < 45%	35 (23.8)	46 (34.6)	**0.047**	9 (26.5)	37 (37.4)	0.249	0.744	**0.022**
Aortic location	87 (58.8)	67 (49.6)	0.123	17 (48.6)	50 (50)	0.884	0.273	0.172
Mitral location	84 (56.8)	92 (68.1)	**0.048**	26 (74.3)	66 (66)	0.365	0.057	0.144
Mitral anterior leaflet	28 (18.9)	28 (20.7)	0.701	8 (22.9)	20 (20)	0.72	0.598	0.834
Mitral posterior leaflet	29 (19.6)	29 (21.5)	0.695	10 (28.6)	19 (19)	0.235	0.244	0.907
Mitral calcified annulus	0	9 (6.7)	**0.001**	1 (2.9)	8 (8)	0.446	0.191	**0.0006**
**Microbiological characteristics**								
Oral Streptococci	32 (21.6)	33 (24.4)	0.573	13 (37.1)	20 (20)	0.042	0.055	0.758
Group D Streptococci	20 (13.5)	13 (9.6)	0.309	3 (8.6)	10 (10)	1	0.576	0.405
*Streptococcus pyogenes*	9 (6.1)	10 (7.4)	0.656	3 (8.6)	7 (7)	0.719	0.703	0.773
Enterococci	18 (12.2)	10 (7.4)	0.181	2 (5.7)	8 (8)	1	0.374	0.294
*Staphylococcus aureus*	33 (22.3)	43 (31.9)	0.07	5 (14.3)	38 (38)	**0.01**	0.293	**0.007**
Coagulase negative staphylococci	9 (6.1)	6 (4.4)	0.539	1 (2.9)	5 (5)	1	0.69	0.717

† Left ventricular ejection fraction

Note: p1: statistical comparison of patients with no neurological complications versus patients with any type of neurological complications; p2: statistical comparison of patients with asymptomatic versus symptomatic neurological complications; p3: statistical comparison of patients without neurological complications versus patients with asymptomatic neurological complications; p4: statistical comparison of patients without neurological complications versus patients with symptomatic neurological complications

**Fig 1:**
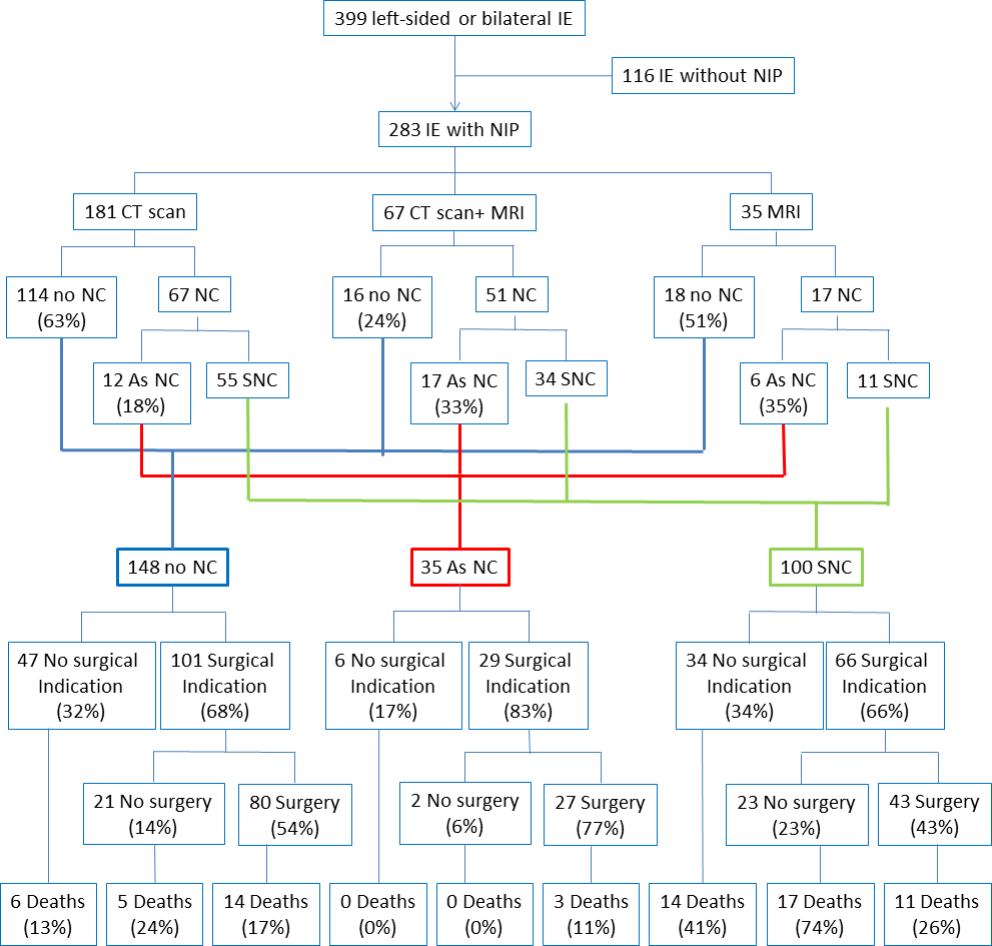
Patients' flow chart. IE: infective endocarditis, NIP: neuroimaging procedure, SNC: symptomatic neurological complications, AsNC: asymptomatic neurological complications, NoNC: no neurological complication.

Among the 283 patients with NIP, 181 patients had only a CT scan, 35 had only a MRI and 67 had both MRI and CT scan (Fig 1). Among the 67 patients with both NIP, 37 had both abnormal CT scan and MRI and 30 had normal CT scan, of whom 19 had abnormal MRI with evidence of ischemic stroke in 18 and of a very small hemorrhage in 1 patient. Among the 248 patients who had a CT scan, it was normal in 130 patients, and revealed a NC in 118 patients, of which 29 (12%) were asymptomatic. Among the 102 patients who had a MRI, it was normal in 34 patients and revealed a NC in 68 patients, of which 23 were asymptomatic (22.5%). AsNC patients had MRI more often than SNC patients (23/35, 65.7% vs 45/100, 45.0%; p = 0.03).

As a whole, 100 patients presented with at least one symptomatic neurological complication (SNC group), 35 patients formed the AsNC group and 148 had normal NIP and constituted the NoNC group (Fig 1).

### Characteristics of patients with neurological complications

Among the 135 patients with NC, 29 patients had more than one type of complication. The distribution of the different types of NC is displayed on Table 2.

**Table 2:** Distribution of the different types of neurological complications among the whole population and among patients with symptomatic and asymptomatic neurological complications.

	Whole population (n = 283) * (% among whole population)	Patients with symptomatic neurological complications (SNC) (n = 100) (% among each type of neurological event)	Patients with asymptomatic neurological complications (AsNC) (n = 35) (% among asymptomatic events)
Ischemic stroke	98 (34.6%)	68 (69.4%)	30 (85.7%)
Transient ischemic attack	9 (3.1%)	9 (100%)	0
Cerebral hemorrhage	33 (11.7%)	26 (78.8%)	7 (20.0%)
Meningitis	17 (6.0%)	17 (100%)	0
Brain abscess	13 (4.6%)	10 (76.9%)	3 (8.6%)
Mycotic aneurysm	9 (3.1%)	4 (44.4%)	5 (1.7%)
Non-specified cerebral event	4 (1.4%)	4 (100%)	0

* 100 symptomatic patients, 35 asymptomatic patients, and 148 patients without neurological complications not represented here.

**Fig 2:**
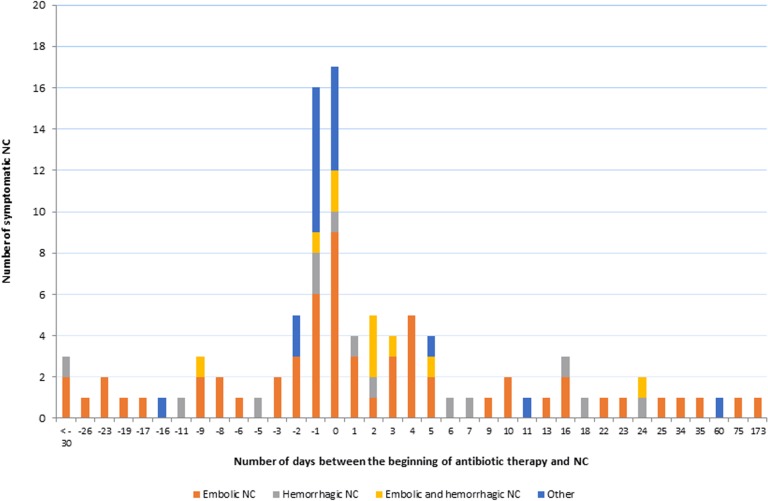
Daily rate of the different types of neurological complications.

Among the 100 patients with symptomatic NC, 40 episodes occurred before the beginning of IE antibiotic therapy, 17 on the same day and 43 after, of which 22 (51%) occurred during the first 5 days (Fig 2).

### Comparison of patients according to their neurological status

The characteristics of different patient subgroups are summarized in Table 1.

Patients with any NC (SNC+AsNC) had a higher rate of prosthetic IE, of embolic events other than cerebral, of mitral location (especially if located on calcified mitral annulus), of septic shock and of elevated CRP, a longer size of vegetation, lower left ventricular ejection fraction, and a lower rate of significant valvular regurgitation than those with NoNC.

Patients with SNC had an older age, a higher rate of elevated CRP, a lower rate of significant valvular regurgitation and a higher rate of IE due to *Staphylococcus aureus* than those with AsNC

Patients with AsNC were younger and had a higher rate of community-acquired IE, of other embolic events and of intracardiac abscess than those with NoNC

### Surgical indications and management

A theoretical surgical indication was reported in 196 (69.2%) patients: 101 NoNC (68.2%), 29 AsNC (82.8%) and 66 SNC (66.0%) patients (Fig 1).

Among these 196 patients, the type of indication was different among patients with and without NC, being significantly more often related to the prevention of embolic event in patients with NC (38% (38/100) of NoNC, 61% (17/28) of AsNC, 80% (51/64) of SNC patients with a specified surgical indication) and more often related to hemodynamic consequences of IE in patients without NC (Table 3).

**Table 3:** Comparison of surgical therapy among patients according to the absence or the presence of neurological complications (NC) either asymptomatic or symptomatic.

	No NC (NoNC) N = 148	Any NC n = 135	p1	Asymptomatic NC (AsNC) n = 35	Symptomatic NC(SNC) n = 100	p2	p3 (NoNC vs AsNC)	p4 (NoNC vs SNC)
**Valvular surgery**	80 (54.1)	70 (51.9)	0.711	27 (77.1)	43 (43)	**0.0005**	**0.013**	**0.088**
**Surgical plan**			0.612			**0.002**	**0.043**	**0.124**
Surgery performed	80 (54.1)	70 (51.9)		27 (77.1)	43 (43)			
No surgery despite surgical indication	21 (14.2)	25 (18.5)		2 (5.7)	23 (23)			
No surgery and no indication	47 (31.8)	40 (29.6)		6 (17.1)	34 (34)			
**Type of surgical indication** (percentages of pts with an indication)			**<0.0001**			0.1015	**0.0438**	**<0.0001**
Hemodynamic	34 (34)	11 (12.0)		5 (17.9)	6 (9.4)			
Infectious	10 (10)	7 (7.6)		2 (7.1)	5 (7.8)			
Embolic	13 (13)	15 (16.3)		1 (3.6)	14 (21.9)			
Hemodynamic + infectious	18 (18)	6 (6.5)		4 (14.3)	2 (3.1)			
Hemodynamic + embolic	14 (14)	18 (19.6)		7 (25)	11 (17.2)			
Infectious + embolic	7 (7)	20 (21.7)		4 (14.3)	16 (25)			
Hemodyn + infectious + embolic	4 (4)	15 (16.3)		5 (17.9)	10 (15.6)			
Missing	1	3		1	2			
**Surgery timing** (percentages of operated pts)			**0.009**			0.138	0.674	**0.001**
Emergency	26 (32.5)	32 (45.7)		11 (40.7)	21 (48.8)			
Elective	52 (65)	30 (42.9)		15 (55.6)	15 (34.9)			
Delayed (temporary contra-indication)	2 (2.5)	8 (11.4)		1 (3.7)	7 (16.3)			
**Time from diagnosis to surgery** (days)	16.1±17.1	12.7±15.7	0.121	9.5±7.4	14.7±19.0	0.9277	0.2088	0.210

Note: p1: statistical comparison of patients with no neurological complications versus patients with any type of neurological complications; p2: statistical comparison of patients with asymptomatic versus symptomatic neurological complications; p3: statistical comparison of patients without neurological complications versus patients with asymptomatic neurological complications; p4: statistical comparison of patients without neurological complications versus patients with symptomatic neurological complications

Cardiac surgery was performed in 150 patients (53.0%): 80 in noNC (54%), 27 in AsNC (77%) and 43 in SNC (43%) (p = 0.0005). Among patients who were operated on, surgery was more often performed either as an emergency measure or postponed due to temporary contra-indication in patients with NC than in patients without NC who more often underwent elective procedure (Table 3). Reasons why surgery was not performed in the 25 patients with NC and theoretical surgical indication were the following: poor general health status (n = 9), early death (n = 5), neurological status (n = 4), disappearance of the vegetation before surgery (n = 1) and improvement under medical therapy (n = 1).

### Mortality and prognostic factors

**Fig 3:**
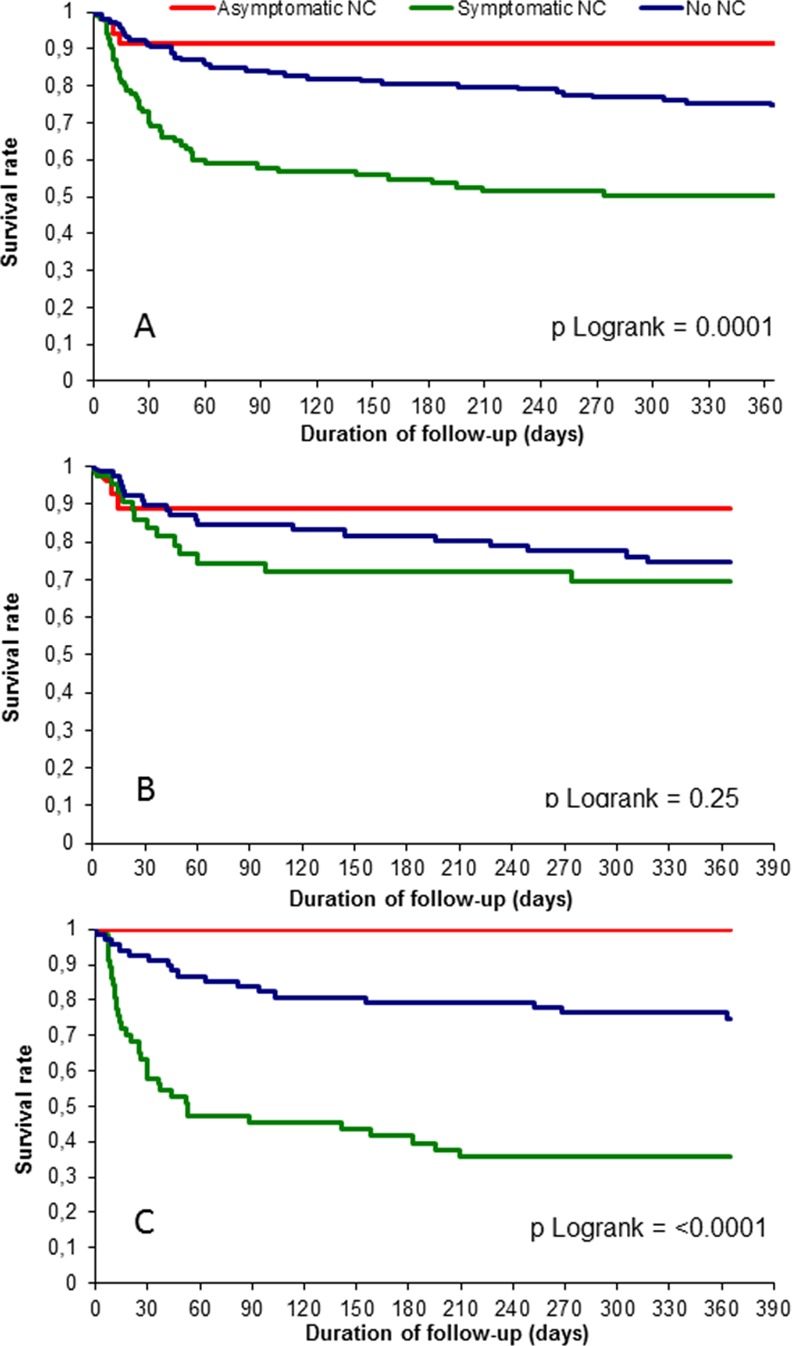
One-year survival curves of A) all patients with neuroimaging procedures (n = 283), B) operated patients (n = 150), C) non operated patients (n = 133) according to the presence and the type of neurological complications.

One third of the patients with SNC had significant neurological sequelae at the end of hospital stay. In-hospital and 1-year mortality rates were respectively 16.9% / 24.3% in NoNC group, 8.6% / 8.6% in AsNC group and 42% / 49% in SNC group (Table 4). Kaplan-Meier curves are displayed in Fig 3 and show a significantly better prognosis of patients with AsNC than patients without NC, the worst prognosis being that of patients with SNC. This was also the case when considering non-operated patients but no longer when considering only operated patients (Fig 3). Among patients with NC, mortality of patients who were operated on was lower than that of patients who had an indication for surgery but who were not operated on.

**Table 4:** Comparison of mortality among patients according to the absence or the presence of neurological complications (NC) either asymptomatic or symptomatic.

	No NC (NoNC) N = 148	Any NC n = 135	p1	Asymptomatic NC (AsNC) n = 35	Symptomatic NC (SNC) n = 100	p2	p3 (NoNC vs AsNC)	p4 (NoNC vs SNC)
**In-hospital mortality**	25 (16.9)	45 (33.3)	**0.001**	3 (8.6)	42 (42)	**0.0003**	0.219	**<0.0001**
Among operated patients	14/80 (17.5)	14/70 (20)	0.695	3/27 (11.1)	11/43 (25.6)	0.141	0.552	0.288
Among non operated patients with indication	5/21 (23.8)	17/25 (68.0)	**0.003**	0/2 (0.0)	17/23 (73.9)	0.093	1	**0.0009**
Among non operated patients without indication	6/47 (12.8)	14/40 (35.0)	**0.014**	0/6 (0.0)	14/34 (41.2)	0.074	1	**0.0034**
**1 year mortality**	36 (24.3)	52 (38.5)	**0.01**	3 (8.6)	49 (49)	**<0.0001**	**0.041**	**<0.0001**
Among operated patients	19/80 (23.8)	16/70 (22.9)	0.897	3/27 (11.1)	13/43 (30.2)	0.064	0.160	0.435
Among non operated patients with indication	7/21 (33.3)	17/25 (68.0)	**0.019**	0/2 (0.0)	17/23 (73.9)	0.093	1	**0.007**
Among non operated patients without indication	10/47 (21.3)	19/40 (47.5)	**0.010**	0/6 (0.0)	19/34 (55.9)	**0.021**	0.581	**0.001**

Note: p1: statistical comparison of patients with no neurological complications versus patients with any type of neurological complications; p2: statistical comparison of patients with asymptomatic versus symptomatic neurological complications; p3: statistical comparison of patients without neurological complications versus patients with asymptomatic neurological complications; p4: statistical comparison of patients without neurological complications versus patients with symptomatic neurological complications

Among the 80 patients with normal NIP who underwent surgery, 2 patients had post-operative new neurological symptoms that quickly resolved: NIP revealed multiple new small ischemic strokes in one patient and a small intracranial hematoma probably related to mycotic aneurysm rupture in the other one.

Among the 70 patients with NC who underwent surgery, 5 died from NC (2 with major cerebral hemorrhages, 1 with multiple new embolic events, 1 with large ischemic stroke pre-existing before surgery and 1 from multiple organ failure together with cerebral abscess complicating initial stroke) and 9 died from another cause. Among survivors, 11 out of 56 (19%) patients had neurological sequelae, but only one patient's neurological status worsened after surgery with an increased size of initial ischemic stroke after surgery.

When adjusted for surgery, SNC was a significant prognostic factor with an OR of 2.7 [95% CI: 1.6–4.5] for in-hospital mortality and of 2.5 [95% CI: 1.6–3.8] for one year mortality. Surgery had a protective effect, but AsNC was no longer predictive of prognosis (Table 5).

**Table 5:** Relationship between neurological complications and mortality among 283 patients with neuroimaging procedure: multivariate analysis adjusted for surgery.

	In-hospital mortality			1-year mortality		
	HR*	95% CI	p value	HR	95% CI	p value
NC**			<0.0001			<0.0001
No NC** (reference)	1			1		
Asymptomatic NC**	0.591	0.177–1.971	0.3925	0.393	0.120–1.281	0.1212
Symptomatic NC**	2.698	1.631–4.461	0.0001	2.499	1.623–3.849	< .0001
Surgery (yes vs no)	0.523	0.321–0.853	0.0094	0.584	0.380–0.898	0.0142

* Hazard ratio

** Neurological complications

Among patients with NC (n = 135), age, renal failure, septic shock and *S*. *aureus* IE were predictive of in-hospital and 1-year mortality (Table 6). Furthermore, SNC was also predictive of 1-year mortality.

**Table 6:** Factors associated with in-hospital and 1-year mortality among patients with neurological complication.

	In-hospital mortality			1-year mortality		
	HR*	95% CI	p value	HR	95% CI	p value
Age (per 1 year)	1.05	1.02–1.07	0.0002	1.06	1.03–1.08	<0.0001
Serum creatinin > 180 μmol/L	2.46	1.31–4.60	0.005	3.39	1.85–6.22	<0.0001
*Staphylococcus aureus*	2.45	1.26–4.75	0.008	2.24	1.17–4.29	0.02
Septic shock	2.34	1.23–4.45	0.009	2.06	1.11–3.84	0.02
Symptomatic NC**				6.51	1.54–27.47	0.01
LVEF† < 45%				1.9	1.06–3.42	0.03

* Hazard ratio

** Neurological complications

† Left ventricular ejection fraction

## Discussion

This observational study describes the incidence, the characteristics and the management of both SNC and AsNC in a large cohort of left-sided IE patients with systematic neuroimaging procedures. The prognosis of patients with SNC was found to be poor and significantly worse than that of patients with AsNC. Furthermore, the prognosis of patients with AsNC was even better than that of patients without NC. In parallel, the rate of surgery was the highest in patients with AsNC, with a high rate of indication related to the prevention of an embolic event. The combination of these results suggests a protective role of surgery guided by systematic neuroimaging results in AsNC.

### Neuroimaging procedures

The percentage of patients with SNC ranges from 12% to 35% in the literature depending on the definition used and is in accordance with the 25% rate in our population-based study.[3–5,9–11] Although not explicitly recommended in the European Society of Cardiology previous guidelines which only stated that "systematic abdominal and cerebral CT scan may be helpful",[2] CT scan or MRI are common and now recommended diagnostic procedures in patients with IE, even in the absence of neurological symptoms. In the 2008 French survey on IE, (8) as much as 70% of the patients with left-sided IE had a NIP, of whom 65% had it in the absence of any neurological symptom. The aim of these systematic imaging procedures is to assess the existence of asymptomatic complications of IE, which may both support the diagnosis and modify the therapeutic strategy. The visualization of large vegetations often leads practitioners to perform such examinations. Indeed, in the 2008 French survey on IE, patients with vegetations longer than 15 mm more often had a NIP than patients with no or with small (< 5 mm) vegetations (74% vs 61%).

The percentage of abnormal findings varies depending on the type of NIP used. Cerebral MRI is superior to CT scan in determining small areas of ischemic and hemorrhagic lesions.[6] Rates of abnormal findings range from only 4% in asymptomatic patients by CT scan [3] to 65–80% by MRI.[5,9] Highly sensitive MRI sequences found abnormalities in 82% of the patients in the IMAGE study, although only 12% were symptomatic.[4] The most frequent lesions were ischemic (37%), and hemorrhagic, from microbleeds to intracerebral hemorrhages. Routine screening by brain CT and CT angiography (CTA) revealed intracranial mycotic aneurysms in 32% of a series of young patients with IE.[12] Our study revealed asymptomatic lesions in 12% of the population when considering both CT scan and MRI, rising to 25% among patients with MRI, which were mostly ischemic (85%). So, our percentage is in complete agreement with the 21% of large ischemic lesions reported in the IMAGE study.

### Neurological complications and surgery

Neurological complications are well known to influence both management and prognosis of IE patients. However, few studies specifically assessed the impact of asymptomatic cerebral lesions. One of our important findings is that AsNC patients are more often operated on than SNC patients and even than NoNC patients. Our rate of 77% is comparable to the 70% rate of surgery in asymptomatic cerebral embolism reported by Thuny.[3] As a comparison, the rate of surgery among patients with symptomatic neurological complications is around 23% in the ICE-PCS cohort [13] and is 43% in our series. As the presence of a previous embolic event associated with large vegetations constitutes a surgical indication, the finding that patients with ischemic NC more often had a surgical indication is logical. In our series, a surgical indication including the prevention of embolism was reported in 50% (38/148) of the patients with NC and in 26% (68/135) of the patients without NC. Furthermore, the rate of no surgery despite the presence of an indication was the lowest in patients with AsNC. Additional factors explaining the high rate of surgery in AsNC patients may include their younger age, a higher frequency of significant valvular regurgitation and abscess as compared to the other patients.

### Neurological complications and prognosis

Another interesting finding of our study is that patients with AsNC had a better one-year survival than those with SNC, but also than those without NC. In the study by Thuny,[3] the 17 patients (4%) with silent cerebral embolism detected by systematic CT scan had a similar death rate than those without NC and a better prognosis than those with SNC. However, this lack of difference of prognosis between AsNC and NoNC patients was more evident after a long period of follow-up (7 years). Notwithstanding, the mortality curves during the first years were in favor of patients with AsNC. Furthermore, as CT scan discovered only 4% of asymptomatic lesions, small silent ischemic events may have been undiagnosed among asymptomatic patients, thus reducing the power to detect statistically significant differences between both groups.

In the light of these results, one might hypothesize that the high rate of surgery in patients with AsNC is responsible for their better prognosis. The results of our multivariate analysis are in agreement with this hypothesis by showing that AsNC does not influence prognosis by itself in opposition to SNC and surgery which have significant and inverse influence on mortality. There has been conflicting results regarding the benefit of surgery in patients with IE depending on the studied population and on the statistical method used.[14] The only randomized study compared early surgery versus conventional therapy (with a 77% rate of surgery) in a highly selected population of young people with streptococcal IE, severe regurgitation and large vegetations. It concluded that early surgery significantly reduced the composite endpoint of death from any cause and embolic events by effectively decreasing the risk of systemic embolism.[15] Of note, in our study, patients with AsNC had the shortest time to surgery, although not significantly so.

The in-hospital and long-term mortality of operated patients was not significantly lower among patients with AsNC than among those with SNC, which is in agreement with the recent study by Misfeld.[16] However, patients with SNC who were operated on had a better prognosis that those who were not operated on, even with no indication. Although these results may be debatable because surgical indications were probably underdiagnosed in those patients due to their neurological status, they suggest that cardiac surgery must always be considered even in symptomatic patients. Our study is therefore a supplementary argument in favor of surgery in patients with embolic risk.

Obviously, indications for systematic imaging methods have to be extended, as their results may considerably change the surgical plan for the patients and perhaps improve their prognosis This is the case in the recent European guidelines which recommend imaging for embolic events in their diagnostic algorithm. [17]

## Limits of the Study

This study reflects the use of NIP in the daily care of patients with IE in France. Although MRI and CT scan are well known to have different diagnostic value, all our patients did not have MRI and some of the patients with normal CT would probably have been classified as abnormal if an MRI had been performed. There may be a selection bias with patients having NIP probably originating from more experienced or tertiary centers; however the comparison between patients with and without NIP did not show significant differences (S1 Table). Furthermore, an initially normal NIP does not preclude the occurrence of asymptomatic NC later on, so some of the patients classified as NoNC could have been AsNC if the NIP had been performed later.

## Conclusion

Neurological events remain a severe complication of IE when they are symptomatic. However, the use of systematic NIP allows early identification of asymptomatic complications which often prompt practitioners towards a more aggressive management with more frequent surgery, and this was associated with a better prognosis in our study. These results, along with those of the only randomized study on surgery in IE,[15] advocate for the clinical importance of early systematic neuroimaging procedure and early use of surgery that are emphasized in the recent guidelines on IE.

## Supporting Information

S1 Table Comparison of general characteristics of the 116 patients with left sided IE without neurological complications and without neuro-imaging procedure versus the 148 patients with left-sided IE without neurological complications and with normal neuro-imaging procedure. (DOCX)

## Abbreviations

AsNC: asymptomatic neurological complication(s)
CT scan: computed tomography scanner
IE: infective endocarditis
MRI: magnetic resonance imaging
NIP: neuroimaging procedure
NC: neurological complication(s)
NoNC: no neurological complication
SNC: Symptomatic neurological complication(s)
