# Supplementary material for: Symptomatic and Asymptomatic Neurological Complications of Infective Endocarditis: Impact on Surgical Management and Prognosis
Source: PLoS One. 2016 Jul 11;11(7):e0158522. doi: 10.1371/journal.pone.0158522 (PMC4939966; doi:10.1371/journal.pone.0158522)
Supplement: S1 Table — (DOCX) [file pone.0158522.s001.docx]

**Supplemental material**

**S1 Table**

|  | **No NIP** N=116 (43.9%) | **Normal NIP** N=148 (56.1%) | p |
| --- | --- | --- | --- |
| **General characteristics** |  |  |  |
| Male gender | 86 (74.1) | 106 (71.6) | 0.649 |
| Age ≥ 60 ans | 83 (71.6) | 87 (58.8) | **0.032** |
| Chronic alcoholism | 12 (10.8) | 18 (12.8) | 0.634 |
| IV drug use | 1 (0.9) | 5 (3.4) | 0.234 |
| Smoking | 19 (17.8) | 32 (23) | 0.313 |
| Charlson score ≥1 | 64 (55.2) | 68 (45.9) | 0.137 |
| Anticoagulation therapy | 35 (30.2) | 38 (25.7) | 0.418 |
| Aspirin therapy | 21 (18.1) | 24 (16.2) | 0.686 |
| **Underlying heart disease** |  |  | 0.117 |
| Native IE | 86 (74.1) | 124 (83.8) |  |
| Prosthetic IE | 30 (25.9) | 24 (16.2) |  |
| **Type of valvular prosthesis** |  |  | 0.153 |
| Mechanical prosthesis | 13 (11.2) | 11 (7.4) |  |
| Bioprosthesis, homograft, autograft | 17 (14.7) | 13 (8.8) |  |
| **Presumed mode of acquisition** |  |  | 0.164 |
| Community | 83 (72.8) | 117 (80.1) |  |
| Health-care associated | 31 (27.2) | 29 (19.9) |  |
| **Complications** |  |  |  |
| Embolic events other than cerebral | 22 (19) | 40 (27) | 0.125 |
| Septic shock (before surgery) | 3 (2.6) | 6 (4.1) | 0.735 |
| **Echocardiographic data** |  |  |  |
| Vegetations | 104 (89.7) | 136 (91.9) | 0.53 |
| *Vegetation size* |  |  | 0.556 |
| ≤ 15 mm | 58 (70.7) | 82 (74.5) |  |
| > 15 mm | 24 (29.3) | 28 (25.5) |  |
| Intracardiac abscess | 21 (18.1) | 26 (17.6) | 0.91 |
| Significant valvular regurgitation | 51 (44.3) | 74 (50) | 0.363 |
| LVEF < 45% | 39 (33.9) | 35 (23.8) | 0.071 |
| **Location of IE** |  |  |  |
| Aortic location | 69 (59.5) | 87 (58.8) | 0.909 |
| Mitral location | 62 (53.4) | 84 (56.8) | 0.592 |
| **Responsible micro-organisms** |  |  |  |
| Oral Streptococci | 20 (17.2) | 32 (21.6) | 0.375 |
| Group D Streptococci | 24 (20.7) | 20 (13.5) | 0.121 |
| Enterococci | 20 (17.2) | 18 (12.2) | 0.243 |
| *Staphylococcus aureus* | 20 (17.2) | 33 (22.3) | 0.309 |
| Coagulase negative staphylococci | 8 (6.9) | 9 (6.1) | 0.789 |
| **Surgery** |  |  | 0.26 |
| Surgery performed | 51 (44) | 80 (54.1) |  |
| No surgery despite surgical indication | 19 (16.4) | 21 (14.2) |  |
| No surgery and no surgical indication | 46 (39.7) | 47 (31.8) |  |
| **Delay of surgery** |  |  | **0.027** |
| Emergency | 28 (54.9) | 26 (32.5) |  |
| Elective | 23 (45.1) | 52 (65) |  |
| Delayed (temporary contra-indication) | 0 (0) | 2 (2.5) |  |
| **Outcome** |  |  |  |
| In-hospital mortality | 23 (19.8) | 25 (16.9) | 0.539 |
| 1-year mortality | 30 (25.9) | 36 (24.3) | 0.775 |

NIP : neuroimaging procedure
